# Obesity and Overweight Among Brazilian Early Adolescents: Variability Across Region, Socioeconomic Status, and Gender

**DOI:** 10.3389/fped.2018.00081

**Published:** 2018-04-06

**Authors:** Chris Fradkin, Nadia C. Valentini, Glauber C. Nobre, João O. L. dos Santos

**Affiliations:** ^1^Department of Psychology, Pontifical Catholic University of Rio de Janeiro, Rio de Janeiro, Brazil; ^2^Institute of Psychology, Federal University of Rio de Janeiro, Rio de Janeiro, Brazil; ^3^Psychological Sciences, University of California, Merced, Merced, CA, United States; ^4^School of Physical Education, Physical Therapy and Dance, Federal University of Rio Grande do Sul, Porto Alegre, Rio Grande do Sul, Brazil; ^5^Federal Institute of Education, Science and Technology of Ceará, Iguatu, Ceará, Brazil; ^6^School of Physical Education and Physical Therapy, Federal University of Amazonas, Manaus, Amazonas, Brazil

**Keywords:** Brazil, obesity, overweight, region, socioeconomic status

## Abstract

**Introduction:**

As with most emerging nations, Brazil lacks up-to-date data on the prevalence of obesity and overweight among its children. Of particular concern is the lack of data on children in early adolescence, considered by many to be the crucial stage for weight-related healthcare.

**Objective:**

To assess regional, socioeconomic, and gender differences in the prevalence of obesity and overweight among Brazilian early adolescents.

**Methods:**

A cross-sectional study was conducted on a racially diverse sample of students aged 10–13 years, from schools in three geographic regions (north, northeast, south) (*N* = 1,738). Data on gender, age, race, socioeconomic status (SES), weight, and height were obtained. Weight class was calculated from age- and gender-adjusted body mass index, based on children’s weight and height. Bivariate and multivariable analyses, with *post hoc* tests, were conducted to estimate differences between groups and were corrected for multiple comparisons. Procedures were approved by institutional review boards at study sites.

**Results:**

Analyses revealed a higher prevalence of obesity and/or overweight among: (1) children of higher SES; (2) children in southern Brazil; (3) males; and (4) Black females.

**Conclusion:**

The most salient predictor of weight risk among Brazilian early adolescents is higher SES. This finding is consistent with previous findings of an inverse social gradient, in weight risk, among emerging-nation population groups.

## Introduction

As in most countries around the globe, the prevalence of obesity among adolescents has increased dramatically in Brazil over the past four decades. According to the Instituto Brasileiro de Geografia e Estatística (IBGE) (1), the agency responsible for the official census in Brazil, obesity among Brazilian adolescents has increased by fourteen times for males and almost six times for females over this period. This increase in weight risk poses considerable physical and psychosocial threats to these adolescents, and overloads the system with the increased cost of weight-related illness. Considering the strong tracking effect of teen obesity into adulthood (2, 3), it is to these children’s benefit, and society’s as well, to monitor weight risk in this group. But, monitoring weight risk in adolescents in Brazil presents several challenges.

Unlike the United States, Brazil does not have up-to-date data on obesity and overweight. There are no surveys conducted, such as the National Health and Nutrition Examination Survey (NHANES) (4), that assess the toll obesity is taking on the youth. Rather, regional studies are conducted in Brazil that provide snapshots of the country’s weight-related health (5–8). While one recent Brazilian study (9) provides compelling insights as to regional and gender-related risk factors, it does not account for socioeconomic status (SES) or racial risk factors. Thus, the Brazilian snapshot on adolescent weight risk is partially composed, but incomplete.

From these fragments, we can gather there are higher rates of obesity among Brazilian youth living in the South (10.4%) than among their counterparts living in the Northeast (4.3%) (5). We can also see that Brazilian youth of higher SES have lower odds of meeting physical fitness guidelines than their counterparts of lower SES: OR = 0.36, 95% CI [0.17, 0.75] (10). We see also that overweight/obesity has slightly higher rates among male Brazilian youth (5.8%) than among their female counterparts (4.0%) (1). And we see higher rates of obesity among urban Brazilian youth (5.4%) than among their rural counterparts (3.0%) (1).

Internationally, there is substantial commitment to reducing obesity and overweight among pediatric populations (11–13). As somewhere between 20 and 80% of obese adolescents become obese adults (14, 15), treating obesity in adolescence has benefits in reducing future weight-related health care in adulthood. The period of early adolescence (10–13 years) is when children establish weight-related behaviors that carry through from adolescence into adulthood (16–18). For this reason, we will focus on this window.

Our aim, therefore, is to examine the prevalence of obesity and overweight among a racially diverse sample of early adolescents, from three geographic regions in Brazil (north, northeast, south). We will examine the relationship between obesity and overweight and region, gender, race, SES, and population density. Based on existing literature, we hypothesize that: (a) There will be a higher prevalence of obesity and overweight among children from the south vs. north and northeast regions; (b) there will be a higher prevalence of obesity and overweight among children of higher vs. lower SES; (c) there will be a slightly higher prevalence of obesity and overweight among males vs. females; and (d) there will be a higher prevalence of obesity and overweight among children living in urban vs. rural environments. As literature is inconsistent on its findings about race, we will address race in an exploratory fashion. As literature is also inconsistent on gender interactions, we will explore these possibilities, with gender nested within the demographic variables.

## Materials and Methods

Data were collected from participants in 10 cities (Acopiara, Cachoeirinha, Camocim, Crato, Florianópolis, Iguacu, Manaus, Porto Alegre, Quezada, and Ubajar), within four states (Amazonas, Ceará, Rio Grande do Sul, and Santa Catarina), within three main regions of Brazil (north, northeast, and south), over a 3-year period (2014–2016). The data were part of a cross-sectional epidemiological study that focused on motor development and its associated risk factors.

### Participants

Study information was presented to boards of education at schools in the 10 participating cities; 16 schools agreed to participate in the study. Procedures were approved by the institutional review boards at the Federal Universities of Rio Grande do Sul, Santa Catarina, and Amazonas, and by the Federal Institute of Ceará; as well as the school boards in the 10 participating cities. Upon approval of procedures, school staff disseminated information to the parents (or caregivers) of the children, which was a sampling pool of approximately 3,200 students. Of this pool, approximately 2,500 students (78%) expressed interest and were sent informed consent forms. Of this group, approximately 1,800 students (72%) returned signed informed consent forms. Exclusion criteria were having a history or known diagnosis of physical disability, or not being in the age range of 10–13 years. This resulted in a final sample of 1,738 participants, with a distribution of 4% Black, 8% Indigenous, 41% Pardo (mixed-race), and 47% White, with 51% females, and a mean youth age of 10.9 years (SD = 1.11). Participants attended public and private schools. Monthly family income ranged from 2 to 15 times federal minimum wage (~210 US dollars/monthly). The sample was composed, primarily, of children from families of lower SES.

### Procedures

This research was conducted in compliance with ethical committee standards in the treatment of participants and was approved by four university institutional review boards and school boards in the participating cities. Anthropometric measurements of the participants were obtained by trained examiners, at the universities or local schools. Demographic information on participants’ family income and socioeconomic characteristics had been gathered by school staff at the beginning of the school year, and was released to researchers through consent of participating families.

### Outcome Variable

Body mass index (BMI) was calculated from measurements of weight and standing height according to standard anthropometric protocols (19) by trained examiners. Standing height was recorded in bare feet or socks, with any hair ornaments the child was wearing removed. Recording was to the nearest millimeter using a portable stadiometer. Weight was recorded to the nearest 0.1 kg using an electronic digital scale, with any heavy clothes the child was wearing removed. Calibration of the scale was checked frequently. Height and weight were used to calculate BMI according to the Quetelet Index: weight (kg)/height (m^2^). Sex-specific ratios of children’s weight for height by age were calculated according to Centers for Disease Control and Prevention guidelines (20), with obese weight class defined as BMI ≥95th percentile and overweight as BMI 85 < 95th percentile.

### Grouping Variables

Race was indexed by local administrators at each site, based on child-report. But, because racial identity among Brazilian children is malleable across developmental stages (21), particularly among Pardo and Black children (22), this initial child-report was supplemented by input from parents and examiners. This format is consistent with Brazilian governmental recommendations that for children under 16 years of age, declaration of race should be provided by adults (23). In cases of disagreement between the initial report and examiner assessment, a second examiner would offer final input. With this system, participants were classified as Black, Indigenous, Pardo, or White.

Socioeconomic status was indexed by federal government protocol (1), based on monthly family income. School characteristics were also considered in our calculation. This resulted in five categories: (1) Very low SES: children from families with monthly household income per capita ≤ twice minimum wage, attending public schools in poor neighborhoods located on the periphery of a city or in a rural region; (2) Low SES: children from families with monthly household income per capita of two-to-three times minimum wage, attending public schools in poor neighborhoods located in the central area of a city; (3) Middle SES: children from families with monthly household income of three-to-twelve times minimum wage, attending public schools located in middle class neighborhoods in the central area of a city; (4) High SES: children from families with monthly household income of twelve-to-fifteen times minimum wage, attending private schools with a tuition >minimum wage, located in upper-class neighborhoods in a city; and (5) Very High SES: children from families with monthly household income >fifteen times minimum wage, attending private schools with a tuition >minimum wage, located in upper-class neighborhoods in a city. As none of the children were in the very high category, the sample was stratified in four levels: very low, low, middle, and high SES.

Population density was indexed by IBGE protocol (24), which was based on municipal laws addressing: urban/rural perimeter, zoning, and the economic contribution of local agribusiness. Based on these criteria, participants were classified as residing in a rural or urban setting. Data on region, school type, and gender were gathered from local school administrative records.

### Data Analysis

Bivariate and multivariable analyses were performed using SPSS version 24.0. Bivariate analyses were conducted to establish prevalences of obesity and overweight for females, males, and both sexes aggregated for each of the three regions, plus for the total sample. This format was repeated for females, males, and both sexes aggregated within the categories of SES, population density, and race. Differences between groups were explored *post hoc* with Wald *F* tests set at *p* < 0.05, using Bonferonni correction for multiple comparisons. Odds ratios (ORs) with confidence intervals (CIs) set at 95% were calculated to express degrees of difference between groups. Significant ORs only are included in results. Next, multivariable analyses were conducted separately for obesity and overweight, with the variables of region, SES, gender, population density, and race included in the model and simultaneously controlling for each other. For significant main effects, differences between groups were explored *post hoc* with Wald *F* tests set at *p* < 0.05, using Bonferonni correction for multiple comparisons.

## Results

### Descriptive Statistics

As shown in Table 1, the majority of the participants were 10 years old, with a fairly even distribution of 11–13 years. Racially, the bulk of the sample was comprised of White and Pardo youth, with a much lower representation of Indigenous and Black youth. Indigenous youth resided in the northern region, while the other racial groups resided in all three (north, northeast, south). Socioeconomically, the bulk of the youth were of low and very low SES, and attended public vs. private school. With regards to population density, all youth in the north- and southern regions were based in urban settings, while youth in the northeast were heterogeneously distributed between rural and urban settings.

**Table 1:** Sample demographics by region.

	Total Sample (*N* = 1,738)		North (*n* = 440)	Northeast (*n* = 712)	South (*n* = 586)
	*n*	%	%	%	%
**Child’s age (years)**					
10	959	55	62	65	38
11	305	18	19	13	22
12	246	14	12	11	19
13	228	13	7	11	21
**Gender**					
Female	881	51	50	47	56
Male	857	49	50	53	44
**Race**					
Black	73	4	7	3	4
Indigenous	142	8	32	0	0
Pardo	712	41	45	63	11
White	811	47	16	34	85
**Socioeconomic status**					
High	94	5	0	13	0
Medium	432	25	0	0	74
Low	817	47	84	41	26
Very low	395	23	16	46	0
**Child’s school**					
Private	95	5	0	13	0
Public	1,643	95	100	87	100
**Population density**					
Rural	303	17	0	43	0
Urban	1,435	83	100	57	100

### Bivariate Analyses

#### Association Between Region and Obesity and Overweight (Hypothesis 1)

As shown in Table 2, among the gender-aggregated sample there were no significant differences in the prevalence of overweight between the different regions. Among gender-specific groups, females from the north had a significantly higher prevalence of overweight than females from the northeast (17 vs. 10% overweight, OR = 1.82, 95% CI [1.11, 2.98]), while there were no significant differences of overweight among males. Among the gender-aggregated sample, children from the south had a significantly higher prevalence of obesity than children from the northeast (9 vs. 6% obesity, OR = 1.79, 95% CI [1.17, 2.74]). Among gender-specific groups, there were no significant differences in obesity prevalence.

**Table 2:** Comparison of overweight and obesity prevalence by region within gender-specific and aggregated groups.

Region	Total% OR [95% CI]	Female% OR [95% CI]	Male% OR [95% CI]
**Overweight**			
Northeast^a^	10.7 1.00	10.4 1.00	10.9 1.00
North	13.9 1.35 [0.94, 1.93]	17.4 **1.82 [1.11, 2.98]**	10.4 0.94 [0.55, 1.62]
South	10.6 0.99 [0.69, 1.41]	12.5 1.23 [0.76, 1.99]	8.1 0.72 [0.42, 1.25]

**Obese**			
Northeast^a^	5.5 1.00	4.8 1.00	6.1 1.00
North	8.4 1.58 [0.99, 2.53]	5.0 1.06 [0.48, 2.34]	11.7 2.04 [1.13, 3.66]
South	9.4 **1.79 [1.17, 2.74]**	8.0 1.73 [0.91, 3.28]	11.2 1.94 [1.09, 3.43]

*Boldface indicates a significant difference between groups after post hoc correction for multiple comparisons. OR, odds ratio; CI, confidence interval*.

*^a^Reference group for comparisons between regions*.

#### Association Between SES and Obesity and Overweight (Hypothesis 2)

As shown in Table 3, among the gender-aggregated sample, children of high SES had a significantly higher prevalence of overweight than children of medium, low, and very low SES (high vs. medium SES: 23 vs. 12% overweight, OR = 2.23, 95% CI [1.28, 3.90]; high vs. low SES: 23 vs. 13% overweight, OR = 2.14, 95% CI [1.27, 3.61]; high vs. very low SES: 23 vs. 6% overweight, OR = 4.94, 95% CI [2.62, 9.34]). In addition, children of medium and low SES had a significantly higher prevalence of overweight than children of very low SES (medium vs. very low SES: 12 vs. 6% overweight, OR = 2.21, 95% CI [1.33, 3.69]; low vs. very low SES: 13 vs. 6% overweight, OR = 2.31, 95% CI [1.44, 3.69]). Among gender-specific groups, females of high SES had a significantly higher prevalence of overweight than females of medium and very low SES (high vs. medium SES: 27 vs. 13% overweight, OR = 2.59, 95% CI [1.21, 5.54]; high vs. very low SES: 27 vs. 5% overweight, OR = 6.89, 95% CI [2.61, 18.23]). In addition, females of low SES had a significantly higher prevalence of overweight than females of very low SES (15 vs. 5% overweight, OR = 3.10, 95% CI [1.45, 6.64]). Among males, males of high SES had a significantly higher prevalence of overweight than males of very low SES (20 vs. 6% overweight, OR = 3.75, 95% CI [1.57, 8.93]). Among the gender-aggregated sample, children of high SES had a significantly higher prevalence of obesity than children of medium, low, and very low SES (high vs. medium SES: 17 vs. 6% obesity, OR = 3.08, 95% CI [1.58, 5.98]; high vs. low SES: 17 vs. 9% obesity, OR = 2.19, 95% CI [1.21, 3.95]; high vs. very low SES: 17 vs. 5% obesity, OR = 4.30, 95% CI [2.10, 8.79]). Among gender-specific groups, females of high SES had a significantly higher prevalence of obesity than females of medium and very low SES (high vs. medium SES: 16 vs. 5% obesity, OR = 3.49, 95% CI [1.31, 9.32]; high vs. very low SES: 16 vs. 3% obesity, OR = 5.68, 95% CI [1.71, 18.90]), while males of high SES had a significantly higher prevalence of obesity than males of very low SES (18 vs. 5% obesity, OR = 3.83, 95% CI [1.54, 9.55]).

**Table 3:** Comparison of overweight and obesity prevalence by SES strata within gender-specific and aggregated groups.

SES	Total% OR [95% CI]	Female% OR [95% CI]	Male% OR [95% CI]
**Overweight**			
Very low^a^	5.8 1.00	5.2 1.00	6.3 1.00
Low	12.5 **2.31 [1.44, 3.69]**	14.5 **3.10 [1.45, 6.64]**	10.3 1.72 [0.93, 3.19]
Medium	12.0 **2.21 [1.33, 3.69]**	12.6 2.66 [1.19, 5.94]	11.2 1.89 [0.94, 3.80]
High	23.4 **4.94 [2.62, 9.34]**	27.3 **6.89 [2.61, 18.23]**	20.0 **3.75 [1.57, 8.93]**

**Obese**			
Very low^a^	4.6 1.00	3.2 1.00	5.4 1.00
Low	8.6 1.96 [1.15, 3.34]	6.5 2.10 [0.79, 5.53]	10.8 2.12 [1.11, 4.04]
Medium	6.3 1.40 [0.76, 2.58]	5.1 1.63 [0.57, 4.65]	7.8 1.48 [0.68, 3.24]
High	17.0 **4.30 [2.10, 8.79]**	15.9 **5.68 [1.71, 18.90]**	18.0 **3.83 [1.54, 9.55]**

*Boldface indicates a significant difference between groups after post hoc correction for multiple comparisons. SES, socioeconomic status; OR, odds ratio; CI, confidence interval*.

*^a^Reference group for comparisons between SES strata*.

#### Association Between Gender and Obesity and Overweight (Hypothesis 3)

As shown in Table 4, females had a significantly higher prevalence of overweight than males (13 vs. 10% overweight, OR = 1.35, 95% CI [1.00, 1.82]), while males had a significantly higher prevalence of obesity than females (9 vs. 6% obesity, OR = 1.56, 95% CI [1.09, 2.25]).

**Table 4:** Comparison of overweight and obesity prevalence by gender.

Gender	Total% OR [95% CI]
**Overweight**	
Female^a^	12.9 1.00
Male	9.9 **0.74 [0.55, 1.00]^b^**

**Obese**	
Female^a^	6.0 1.00
Male	9.1 **1.56 [1.09, 2.25]**

*Boldface indicates a significant OR at p < 0.05. OR, odds ratio; CI, confidence interval*.

*^a^Reference group for comparisons*.

*^b^OR referenced to male = 1.35 [1.00, 1.82]*.

#### Association Between Population Density and Obesity and Overweight (Hypothesis 4)

As shown in Table 5, among the gender-aggregated sample, children based in urban settings had a significantly higher prevalence of overweight than children based in rural settings (13 vs. 6% overweight, OR = 2.14, 95% CI [1.31, 3.50]). Among gender-specific groups, females based in urban settings had a significantly higher prevalence of overweight than females based in rural settings (14 vs. 6% overweight, OR = 2.88, 95% CI [1.37, 6.05]), while there were no significant differences of overweight among males. Among the gender-aggregated sample, children based in urban settings had a significantly higher prevalence of obesity than children based in rural settings (8 vs. 3% obesity, OR = 2.70, 95% CI [1.40, 5.21]). Among gender-specific groups, males based in urban settings had a significantly higher prevalence of obesity than males based in rural settings (10 vs. 3% obesity, OR = 3.57, 95% CI [1.42, 8.98]), while there were no significant differences of obesity among females.

**Table 5:** Comparison of overweight and obesity prevalence by population density within gender-specific and aggregated groups.

Population density	Total% OR [95% CI]	Female% OR [95% CI]	Male% OR [95% CI]
**Overweight**			
Rural^a^	6.3 1.00	5.5 1.00	7.0 1.00
Urban	12.5 **2.14 [1.31, 3.50]**	14.4 **2.88 [1.37, 6.05]**	10.6 1.58 [0.82, 3.06]

**Obese**			
Rural^a^	3.3 1.00	3.4 1.00	3.2 1.00
Urban	8.4 **2.70 [1.40, 5.21]**	6.5 1.95 [0.76, 5.00]	10.4 **3.57 [1.42, 8.98]**

*Boldface indicates a significant OR at p < 0.05. OR, odds ratio; CI, confidence interval*.

*^a^Reference group for comparisons*.

#### Association Between Race and Obesity and Overweight (Non-Hypothesized)

As shown in Table 6, among the gender-aggregated sample there were no significant differences in the prevalence of overweight between the different racial groups. Likewise, among the gender-specific groups, there were no significant differences, as well. Among the gender-aggregated sample, there were no significant differences in the prevalence of obesity between the different racial groups. Among gender-specific groups, Black females had a significantly higher prevalence of obesity than Pardo and White females (Black vs. Pardo: 17 vs. 6% obesity, OR = 3.42, 95% CI [1.27, 9.24]; Black vs White: 17 vs. 6% obesity, OR = 3.46, 95% CI [1.32, 9.10]), while among males, there were no significant differences in the prevalence of obesity between the different racial groups.

**Table 6:** Comparison of overweight and obesity prevalence by race within gender-specific and aggregated groups.

Race	Total% OR [95% CI]	Female% OR [95% CI]	Male% OR [95% CI]
**Overweight**			
Pardo^a^	9.7 1.00	11.7 1.00	7.9 1.00
Black	12.3 1.31 [0.63, 2.75]	20.0 1.89 [0.77, 4.60]	5.3 0.65 [0.15, 2.82]
Indigenous	9.9 1.02 [0.56, 1.87]	14.3 1.26 [0.60, 2.66]	5.6 0.68 [0.23, 2.01]
White	13.2 1.42 [1.03, 1.95]	13.1 1.14 [0.74, 1.75]	13.3 1.79 [1.11, 2.89]

**Obese**			
Pardo^a^	6.0 1.00	5.7 1.00	6.1 3.00
Black	13.7 2.47 [1.18, 5.15]	17.1 **3.42 [1.27, 9.24]**	10.5 1.74 [0.57, 5.31]
Indigenous	7.7 1.31 [0.66, 2.60]	4.3 0.74 [0.21, 2.57]	11.1 1.85 [0.80, 4.30]
White	8.3 1.40 [0.94, 2.08]	5.6 0.99 [0.54, 1.83]	11.4 1.91 [1.13, 3.22]

*Boldface indicates a significant difference between groups after post hoc correction for multiple comparisons. OR, odds ratio; CI, confidence interval*.

*^a^Reference group for comparisons between races*.

### Multivariable Analyses

As shown in Table 7, there were significant main effects for region and SES throughout the two analyses. With regards to overweight, *post hoc* analysis indicated a positive relationship between SES and overweight, in which children of high SES had a significantly higher prevalence of overweight than children of medium and low SES, who had a significantly higher prevalence of overweight than children of very low SES (see Table 3 for prevalences). With regards to obesity, *post hoc* analysis indicated that children based in the southern region had a higher prevalence of obesity than their counterparts based in the northeast region (see Table 2 for prevalences). Children of high SES had a significantly higher prevalence of obesity than children of medium, low, and very low SES. And finally, males had a significantly higher prevalence of obesity than females.

**Table 7:** Multivariable analyses for overweight and obesity across sociodemographic variables.

Source	Wald *F*
**Overweight**	
Region	9.75** n.s. *post hoc*
SES	12.72** H > M, L > VL
Gender	–
Population density	–
Race	–

**Obese**	
Region	18.41*** S > NE
SES	32.65*** H > M, L, VL
Gender	4.98* M > F
Population density	–
Race	–

*Only significant main effects and post hoc differences reported; n.s. post hoc, post hoc differences not significant using Bonferonni correction for multiple comparisons set at p < 0.05; > indicates significantly higher weight risk; SES: H, high; M, medium; L, low; VL, very low; region: NE, northeast; S, south; gender: F, female; M, male*.

*p < 0.05. **p < 0.01. ***p < 0.001.

## Discussion

This study indicates that there is considerable variability in the prevalence of obesity and overweight among Brazilian early adolescents. Partially consistent with Hypothesis 1 is the bivariate and multivariable finding of a lower prevalence of obesity among those children in the northeast, as compared to children in the south. Consistent with Hypothesis 2 is the bivariate and multivariable finding of a higher prevalence of obesity and overweight among children of higher SES, as compared to children of lower socioeconomic strata. Partially consistent with Hypothesis 3 is the bivariate and multivariable finding of a higher prevalence of obesity among males, as compared to females. Partially consistent with Hypothesis 4 is the bivariate finding of a higher prevalence of obesity and overweight among children based in urban settings, as compared to children based in rural settings. And finally, there was also a bivariate finding of a higher prevalence of obesity among Black females, as compared to their White and Pardo female counterparts. As these last two findings were not supported in the multivariable analysis, this suggests that interactions may be present, although addressing them was beyond the purview of this paper.

Consistent with the Niehues and colleagues study (5) is the finding of a lower prevalence of obesity among children in the northeast, than children in the south. To understand this fully, we must consider the makeup of these regions. As seen in Table 1, there is a higher concentration of children from families of very low and low SES groups in the northeast (87%) than children in the south (26%). The northeast region of the country is considerably poorer than the south, and a sizeable portion (43%) of this region is rural. In sum, rural vs. urban, and lower vs. higher SES, appear protective against weight risk among Brazilian children.

These differences are backed by recent studies (10, 25, 26). For one, Matsudo and colleagues (10) found higher SES associated with lower odds of children meeting physical fitness guidelines, OR = 0.36, 95% CI [0.17, 0.75]. The authors also found household auto ownership associated with lower odds of children meeting guidelines, OR = 0.48, 95% CI [0.31, 0.75]. With regards to diet, da Costa Louzada and colleagues (25) found the consumption of high-fat, high-caloric ultra-processed foods (e.g., ice-creams, soft drinks, industrialized baked products) higher among children in urban vs. rural areas. A separate study (26) on the availability of these foods found them more present in the homes of higher SES, urban families than in the homes of their lower SES, rural counterparts. And there are further aspects to consider. As the northeast region is much poorer than the south, northeast families depend more heavily on their children. While the southern child may spend hours on their cell phone after school, their northeast counterpart may be tending younger siblings, or working in the garden after school. These active hours are protective against obesity, as the sedentary hours present risk.

Our findings suggest a positive relationship between SES and weight risk. This is consistent with the review by Dinsa and colleagues (27), which found that child obesity in emerging nations was more prevalent among families of higher SES. This relationship is contrary to the social gradient of weight, in which SES and weight risk are negatively related. In the United States, for example, among a racially diverse sample of early adolescents (*N* = 4,824; M_age_ = 11.12 years), children of very low SES had a higher prevalence of obesity than those of high SES (31 vs. 19% obesity, OR = 1.95, 95% CI [1.53, 2.48]) (16). In addition, the mean obesity prevalences of the samples bears attention. In the study from the United States (16), the mean obesity prevalence is 27%; in this study the same prevalence is 8%. And in this study, at its most extreme—among males of high SES—the obesity prevalence is 18%. This 18% prevalence is lower, by the way, than the prevalence of the healthiest subgroup in the United States study: children of high SES (19% obesity). For Brazilians, this contrast may be heartening: meaning the epidemic has yet to reach its full potential. Nonetheless, obesity is threatening. With increased fast-food access, with lifestyles sedentary, Brazilians of all ages are at risk.

Though more modest in their nature, other findings from our study should be mentioned. While higher obesity among males is consistent with several findings (1, 28), its contribution to the model is the weakest: Wald *F* = 4.98, *p* = 0.03 (see Table 7). Of greater interest is the bivariate finding of higher obesity among Black females (17%), as compared to their Pardo (6%) and White (6%) counterparts (see Table 6). This relationship reflects the findings of a study from the United States (29). In that study of weight risk among an early adolescent sample (*N* = 4,824; M_age_ = 11.11 years), Black females had a significantly higher prevalence of obesity (32%) than their same-sex White counterparts (15%), OR = 2.28 [1.40, 3.72]. These parallel findings may suggest a racial contribution to obesity that is independent of culture and location.

With regards to future research, future studies should examine interactions between the predictors we examined (e.g., region × SES, race × SES, gender × region) and weight risk among Brazilian youth. Research should also consider third variables as well (e.g., fast-food restaurant density, availability of fresh produce, neighborhood availability of safe areas for physical activity), and their relationship with obesity in youth. As early adolescence is a stage crucial to weight-related healthcare (30), future studies would be wise to focus on this stage: on youth of every region, culture, race, and tribe, etc., as defined by gender, neighborhood, and social strata. But for now, we turn back to the present study.

This study is the first we are aware of, on a Brazilian population, that has examined pediatric weight risk in relation to multiple sociodemographic factors, using a multivariable approach. This approach reveals relationships between the variables of interest, with the different variables controlling for each other. The result is an integrated snapshot not available in previous studies on the subject.

Among limitations in the study: first is the demographic balance. As shown in Table 1, while there is a balanced proportion of Pardo and White children (41 and 47%, respectively), there is an underrepresentation of Black and Indigenous children (4 and 8%, respectively). This underrepresentation, especially among Black children, may have contributed to the paucity of main effects of race within the study. Socioeconomically, there is also an underrepresentation of children in the high SES strata (5%), an anomaly that makes the higher prevalence of weight risk in this group even more striking.

Another limitation of the study is its restricted distribution, which sampled children from 10 cities in three regions. This distribution, as opposed to data from all regions, limits generalizations of the findings nationwide. And finally, as with all correlational studies, we are limited in our inference of causality. Nonetheless, it is less likely that obesity and overweight impacted SES, race, and gender than these factors had an influence on weight risk.

But limitations notwithstanding, this study has implications for healthcare, policy, and research. One implication is that weight risk among early adolescents in Brazil is the most threatening to children of higher socioeconomic means. As the caretakers of this group are of a higher educational and financial/social stratum than their less stable and less affluent counterparts, one might infer they would be receptive to family interventions that aim to modify weight-related behaviors. Future studies could track the efficacy of weight-related family interventions, as the children mature over time. Future studies could also employ geospatial analysis to map fast-food availability and its relationship to weight risk within sectors. Geospatial analysis could also monitor the availability of fresh produce and fresh fruit, and safe zones for exercise, and their relationships to weight risk within sectors. With regards to policy, our aim is that these findings set dialog in motion that prompts large-scale surveys on weight risk among children.

## Conclusion

In order to better understand the threat of obesity among children, Brazil must prioritize data collection and research on this group. Of particular importance are early adolescents, who are at a crucial period for weight-related healthcare (30). While this study confirms findings from recent research (5, 8, 9, 31, 32), it is our hope that it will lead to further steps. Large-scale surveys are now needed, and data from them for our research, to lay foundation for future healthcare policy.

## Ethics Statement

This study was carried out in accordance with the recommendations of the Federal Universities of Brazil with written informed consent from all subjects. The protocol was approved by the institutional review boards at the Federal Universities of Rio Grande do Sul, Santa Catarina, and Amazonas, and by the Federal Institute of Ceará; as well as the school boards in the 10 participating cities.

## Author Contributions

CF and NV conceived the study, conducted the analyses, interpreted the data, and wrote the paper. NV collected the data. GN and JS reviewed and critically revised the manuscript. All authors read and approved the final manuscript.

## Conflict of Interest Statement

The authors declare that the research was conducted in the absence of any commercial or financial relationships that could be construed as a potential conflict of interest.
